# Atmospheric Influences Controlling Inebriety

**Published:** 1877-09

**Authors:** T. D. Crothers

**Affiliations:** Superintendent of Walnut Hill, Hartford, Conn.


Atmospheric Influences Controlling Inebriety.—*By T. D. Crothers, M.D., Superintendent of Walnut Hill, Hartford, Conn.* At the Philadelphia meeting of the Association for the Cure of Inebriates, I reported a curious fact, that periods of great restlessness and irritability so common among inebriates under treatment, seemed to follow the rapid fluctuations of the barometer, and be particularly marked, in low areas of atmospheric depression.

At that time my observations had been confined to a short period, and the statement made seemed to be indicated, although not confirmed, by sufficient evidence. I propose now to state some of the observations which have been noted, as a confirmation of the above statement. All persons who have charge of inebriates and note the progress of the case from day to day, are surprised at the exceedingly variable character of the disorder generally manifested in extremes of physical and mental debility. Noted as either abject invalids, craving for medicine and help, or ignoring all efforts and boasting of conscious strength, accompanied with periods of restlessness and excitement from trivial causes, which come and go rapidly.

These paroxysms seem to appear at all times and pervade all classes of inebriates, from the patient lately arrived and not over the immediate effects of alcohol, to those who have been for months residents of the asylum. My attention was called to the similarity of these paroxysms, and their frequent returns, without any apparent cause.

This led me to note down carefully all the symptoms, which may be described in a general way, as follows: Without premonition or apparent cause the patient becomes restless and exhibits a tendency to boisterous emotional displays. Patients will walk up and down the halls or grounds, or desire to go away on long tramps, without any particular purpose, and laugh loudly and immoderately at trivial things; show great irritability and anger at insignificant objects; or manifest in extreme sensitiveness and complain bitterly of their conditions and surroundings. The appetite seems to decline, and a half defined stage of general depression comes on, attended with an intense longing for medicines of any kind; various neuralgic affections appear, accompanied with pains of all kinds, headaches, dyspepsia, sense of weight and fullness. A degree of recklessness and abandon are manifest, rules and healthy restraint are disregarded, and keen pleasure is apparent in detailing drinking scenes, etc.

In appearance they betray agitation and general nervousness; often the flashing eye and the tremulous lips and muscular movements, betoken the coming storm. The mind is free from delusions, although intensely sensitive, and likely to explode in outbursts of indignation, or appeals of generosity, flying from one extreme to another. The management of such cases during these paroxysms is always difficult, requiring rare tact and judgment. The care and attention must be increased, and not unfrequently all the medical and hygeinic measures are taxed to their utmost. Such attacks usually last from twelve to thirty hours, then subside. Frequently these paroxysms end in drunkenness from liquor introduced into the asylum with the greatest adroitness and cunning.

After recording a number of these instances and trying to eliminate all possible complications and coincidents, I was surprised to notice that they seemed to occur just before or during a storm.

Turning to our barometer, whose readings are recorded three times a day, I found that the time of these paroxysms corresponded to the sudden fluctuations of the atmosphere, particularly a falling barometer and low area of pressure. I directed the observer to note with care the sudden changes and low area of pressure, with temperature, winds, etc. While I recorded carefully all the circumstances and conditions that seemed to enter into these paroxysms, each keeping his record distinct, and not under the observation of the other.

Having recorded twenty instances or paroxysms, similar to that described above, extending over seven months, a comparison of records was made, showing that fourteen of these instances occurred at the time the barometer indicated a very low area of pressure, nearly or about 28.30 inches.

In the six remaining instances there was a steady or changing barometer, the mercury in two cases being on the point of fluctuating either way. In six cases, this low barometer continued eighteen or twenty hours, and in the other cases a much less time. The length of the paroxysms seemed to correspond to this long-continued pressure; also cloudy and threatening storm was marked at some of the times recorded. Paroxysm number twelve in my note book was noted as coming on in the afternoon, and attended with unusual depression, extending to nearly every patient, and manifest in a very marked way, clearing up next day.

The record of the barometer at this time indicated a rapid fall and an unexpected low pressure, with an almost equally sudden rise. The range of the barometer was below 28 inches and rose within eighteen hours to 29.26.

It was found that from four to six hours after the barometer began to fall, or reached its lowest area, these paroxysms commenced. The records of the temperature at these times, and the direction of the winds, were not in any way significant. On two occasions a severe thunder storm followed, which was noted in my records by an unusual degree of fault-finding and general disaffection among the patients. I regret that I cannot give a diagram showing the correspondence between these two records, but trust to continue these observations in the future with results more satisfactory. These statements are not presented as conclusive in any way, but rather as hints and suggestions of the possibilities awaiting further investigations.

There is a widespread belief of the influence of the atmosphere over nervous diseases, and superintendents of insane asylums have noted the emotional and other changes appearing in their patients before the occurrence of a storm. Dr. S. Weir Mitchell remarks very clearly as follows: "Apart from these outside conditions of weather which we may conceive of as influencing us by checking or increasing perspiration, and lessening surface pressure, or altering the amount of oxygen inspired and carbonic acid thrown out, there are no doubt personal elements in the equation which are more or less mysterious, and which must be taken to account for a certain number of the neuralgic fits which do not seem to have perfect relations to casual weather states; moreover it is conceivable that as we are changeful instruments, the body may be sometimes more liable to respond by pain to conditions of weather favorable to its production.

"The human economy is arranged by nature to have, as it were, a climate of its own, with very permanent states as to temperature, humidity, electric conditions, and the like; but all of these are subject to variations, some of them natural, and, so to speak, rythmic and chronal; others more or less irregular. As they are part of the functional activities of the body, so do they, if necessary, enter into every consideration of the causation of pain. While, however, we may feel sure that they are thus active, their precise relations to the existence or to the favoring of the birth of pain are too uncertain for us to do more than surmise that they sometimes obscure or interfere with or prevent the positive effects of external climatic states in this direction. Any lowering cause, such as dyspepsia, overwork, and anæmia, however brought about, is apt to increase this sensitiveness to barometric changes; and so every enfeebling agency, as it were, tones a man's nerve up to the capacity of producing pain, when once there exists a permanent cause in the way of neural disease." . . . . . .

In an inebriate asylum it is difficult to eliminate all the possible causes which might produce these so-called paroxysms, and limit it to atmospheric influences alone. Inebriates, as a class, are extremely sensitive to all changes of surroundings, and likely to become greatly agitated from the most trifling causes.

The arrival of a patient, very much intoxicated, at the asylum, particularly if he is allowed to stand around and be seen, creates more or less excitement that is dangerous to others, and often awakens diseased cravings for liquor that are incontrollable. At certain times patients will recognize their ill feelings as arising from perverted nutrient desires, and reason clearly about it, seeking relief with earnestness. On other occasions they become secretive and reserved in their manner, exhibiting morbid excitement and want of principle that is abject.

The exercise of wholesome discipline at times creates much sympathetic excitement, and the absence of restraint necessary to check the precipitation of some cases, has the same effect. To eliminate these and other causes, and indicate how far the atmospheric influences control the organism, will require extended observation in many asylums. I have noted several cases where some obscure conditions of the atmosphere, either physical or electrical, seemed to be the exciting cause of inebriety.

In one instance the salt air of the sea shore raises intolerable cravings for liquor; in another, a mountain region and the solitude of the woods, etc.

The influence which I have drawn from these and other observations is that conditions of atmospheric pressure do often exercise a powerful influence over inebriety, and that frequently a change of climate is followed by a lessened desire for stimulants; also it is not improbable that we shall find in the future that certain temperatures, climates, and conditions of the atmosphere are favorable for the continuation of inebriety, or rapid decline, and finally dying away of the disorder.

The influences of climate, changing conditions of weather, winds, fogs, dampness, cold, heat, dense or rarified air, rivers, oceans, soil, and contour of the land, act either to raise or lower the standard of healthy activity; and the reaction of these changes manifest in the nutrient wants, when perverted, constitute the starting point of inebriety.—*Quarterly Journal of Inebriety.*

